# 2-Amino-4-methyl­pyridinium 3-hy­droxy­benzoate

**DOI:** 10.1107/S1600536813016310

**Published:** 2013-06-19

**Authors:** Nuridayanti Che Khalib, Kaliyaperumal Thanigaimani, Suhana Arshad, Ibrahim Abdul Razak

**Affiliations:** a School of Physics, Universiti Sains Malaysia, 11800 USM, Penang, Malaysia

## Abstract

In the title salt, C_6_H_9_N_2_^+^·C_7_H_5_O_3_^−^, the anion is essentially planar, with a dihedral angle of 2.72 (17)° between the benzene ring and the carboxyl­ate group. In the crystal, the anions are connected by O—H⋯O hydrogen bonds, forming a 4_1_ helical chain along the *c* axis. The protonated N atom and the 2-amino group of the cation are hydrogen bonded to the carboxyl­ate O atoms of the anion *via* a pair of N—H⋯O hydrogen bonds with an *R*_2_^2^(8) ring motif. The ion pairs are further connected *via* another N—H⋯O hydrogen bond, resulting in a three-dimensional network.

## Related literature

For the role of hydrogen bonding in crystal engineering, see: Goswami & Ghosh (1997 ▶); Goswami *et al.* (1998 ▶); Lehn (1992 ▶). For related structures, see: Kvick & Noordik (1977 ▶). For hydrogen-bond motifs, see: Bernstein *et al.* (1995 ▶). For bond-length data, see: Allen *et al.* (1987 ▶). For the stability of the temperature controller used in the data collection, see: Cosier & Glazer (1986 ▶).
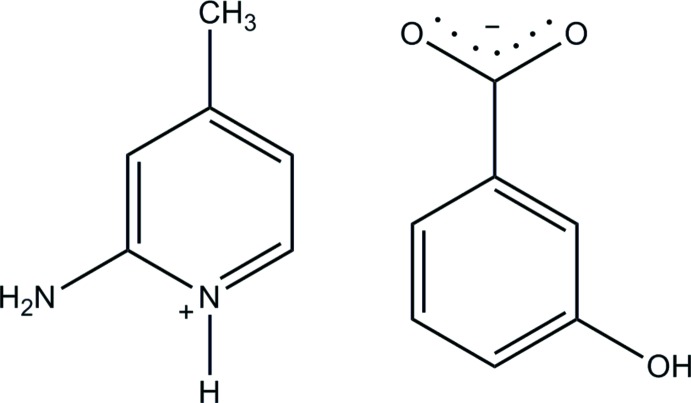


## Experimental

### 

#### Crystal data

C_6_H_9_N_2_^+^·C_7_H_5_O_3_^−^
*M* *_r_* = 246.26
Tetragonal, 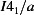

*a* = 15.4435 (2) Å
*c* = 21.0395 (3) Å
*V* = 5017.96 (12) Å^3^
*Z* = 16
Mo *K*α radiation
μ = 0.09 mm^−1^
*T* = 100 K
0.43 × 0.26 × 0.23 mm

#### Data collection

Bruker SMART APEXII CCD area-detector diffractometer
Absorption correction: multi-scan (*SADABS*; Bruker, 2009 ▶) *T*_min_ = 0.961, *T*_max_ = 0.979
46924 measured reflections
3702 independent reflections
3092 reflections with *I* > 2σ(*I*)
*R*_int_ = 0.058

#### Refinement

*R*[*F* ^2^ > 2σ(*F* ^2^)] = 0.060
*wR*(*F* ^2^) = 0.130
*S* = 1.10
3702 reflections
180 parameters
H atoms treated by a mixture of independent and constrained refinement
Δρ_max_ = 0.45 e Å^−3^
Δρ_min_ = −0.30 e Å^−3^

### 

Data collection: *APEX2* (Bruker, 2009 ▶); cell refinement: *SAINT* (Bruker, 2009 ▶); data reduction: *SAINT*; program(s) used to solve structure: *SHELXTL* (Sheldrick, 2008 ▶); program(s) used to refine structure: *SHELXTL*; molecular graphics: *SHELXTL*; software used to prepare material for publication: *SHELXTL* and *PLATON* (Spek, 2009 ▶).

## Supplementary Material

Crystal structure: contains datablock(s) global, I. DOI: 10.1107/S1600536813016310/is5282sup1.cif

Structure factors: contains datablock(s) I. DOI: 10.1107/S1600536813016310/is5282Isup2.hkl

Supplementary material file. DOI: 10.1107/S1600536813016310/is5282Isup3.cml

Additional supplementary materials:  crystallographic information; 3D view; checkCIF report

## Figures and Tables

**Table 1:** Hydrogen-bond geometry (Å, °)

*D*—H⋯*A*	*D*—H	H⋯*A*	*D*⋯*A*	*D*—H⋯*A*
O1—H1*O*1⋯O2^i^	0.89 (2)	1.81 (2)	2.6970 (15)	172 (2)
N1—H1*N*1⋯O2^ii^	0.92 (2)	1.81 (2)	2.7221 (15)	172 (2)
N2—H1*N*2⋯O3^ii^	0.88 (2)	1.91 (2)	2.7852 (16)	170.6 (19)
N2—H2*N*2⋯O3	0.883 (19)	1.994 (19)	2.8454 (17)	161.7 (18)

Symmetry codes: (i) 
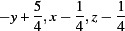
; (ii) 
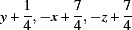
.

**Fig. 1.**
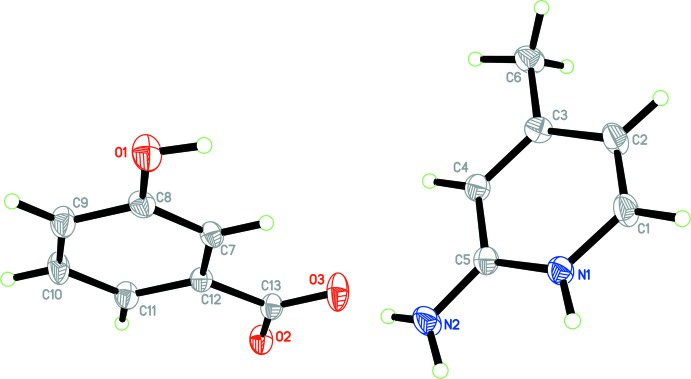
The molecular structure of the title compound with atom labels with 50% probability displacement ellipsoids.

**Fig. 2.**
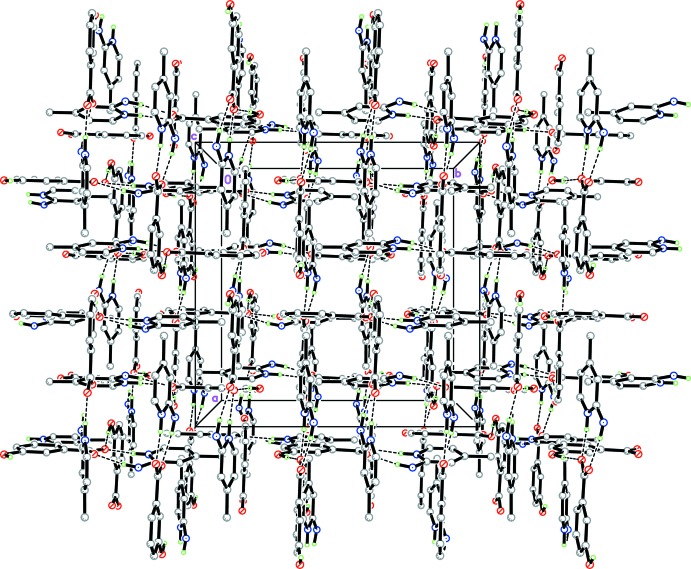
The crystal packing of the title compound. The H atoms not involved in the intermolecular interactions (dashed lines) have been omitted for clarity.

## supplementary crystallographic information

### Comment

Hydrogen bonding plays a key role in molecular recognition (Goswami & Ghosh, 1997) and crystal engineering research (Goswami *et al.*, 1998). The design of highly specific solid-state compounds is of considerable significance in organic chemistry due to important applications of these compounds in the development of new optical, magnetic and electronic systems (Lehn, 1992). In order to study some hydrogen bonding interactions, the synthesis and structure of the title salt, (I), is presented here.

The asymmetric unit (Fig. 1) contains one 2-amino-4-methylpyridinium cation and one 3-hydroxybenzoate anion. A proton is transfered from the carboxyl group to atom N1 of 2-amino-4-methylpyridine, resulting in the widening of C1–N1–C5 angle of the pyridinium ring to 122.25 (12)°, compared to the corresponding angle of 117.3 (1)° in neutral 2-amino-4-methylpyridine (Kvick & Noordik, 1977). The 2-amino-4-methylpyridinium cation is essentially planar, with a maximum deviation of 0.007 (1) Å for atom C5. The carboxylate group of the 3-hydroxybenzoate anion is slightly twisted from the attached ring with a dihedral angle between the C7–C12 ring and the O2/O3/C13 plane being 2.72 (17)°. The bond lengths and angles are normal (Allen *et al.*, 1987).

In the crystal packing (Fig. 2), the anions are connected by O1—H1O1···O2^i^ hydrogen bonds (symmetry code in Table 1). The protonated N1 atom and the 2-amino group (N2) of the cation are hydrogen-bonded to the carboxylate oxygen atoms of the anion (O2 and O3, respectively) *via* a pair of intermolecular N1—H1N1···O2^ii^ and N2—H1N2···O3^ii^ hydrogen bonds (symmetry code in Table 1), forming an *R*_2_^2^(8) (Bernstein *et al.*, 1995) ring motif. These motifs are then connected *via* N2—H2N2···O3 hydrogen bond (Table 1), resulting in a three-dimensional network.

### Experimental

Hot methanol solution (20 ml) of 2-amino-4-methylpyridine (54 mg, Aldrich) and 3-hydroxybenzoic acid (35 mg, Merck) were mixed and warmed over a heating magnetic-stirrer hotplate for a few minutes. The resulting solution was allowed to cool slowly at room temperature and crystals of the title compound (I) appeared after a few days.

### Refinement

O- and N- bound H atoms were located in a difference Fourier map and allowed to be refined freely [O—H = 0.89 (2) Å and N—H = 0.88 (2) and 0.92 (2) Å]. The remaining H atoms were positioned geometrically (C—H = 0.95 or 0.98 Å) and were refined using a riding model, with *U*_iso_(H) = 1.2*U*_eq_(C) or 1.5*U*_eq_(methyl C). A rotating-group model was used for the methyl group. One outlier (0 2 0) was omitted in the final refinement.

### Crystal data

**Table:**

C_6_H_9_N_2_^+^·C_7_H_5_O_3_^−^	*D*_x_ = 1.304 Mg m^−^^3^
*M**_r_* = 246.26	Mo *K*α radiation, λ = 0.71073 Å
Tetragonal, *I*4_1_/*a*	Cell parameters from 9925 reflections
Hall symbol: -I 4ad	θ = 2.6–30.0°
*a* = 15.4435 (2) Å	µ = 0.09 mm^−^^1^
*c* = 21.0395 (3) Å	*T* = 100 K
*V* = 5017.96 (12) Å^3^	Block, colourless
*Z* = 16	0.43 × 0.26 × 0.23 mm
*F*(000) = 2080	

### Data collection

**Table:**

Bruker SMART APEXII CCD area-detector diffractometer	3702 independent reflections
Radiation source: fine-focus sealed tube	3092 reflections with *I* > 2σ(*I*)
Graphite monochromator	*R*_int_ = 0.058
φ and ω scans	θ_max_ = 30.1°, θ_min_ = 1.6°
Absorption correction: multi-scan (*SADABS*; Bruker, 2009)	*h* = −21→21
*T*_min_ = 0.961, *T*_max_ = 0.979	*k* = −21→21
46924 measured reflections	*l* = −29→29

### Refinement

**Table:**

Refinement on *F*^2^	Primary atom site location: structure-invariant direct methods
Least-squares matrix: full	Secondary atom site location: difference Fourier map
*R*[*F*^2^ > 2σ(*F*^2^)] = 0.060	Hydrogen site location: inferred from neighbouring sites
*wR*(*F*^2^) = 0.130	H atoms treated by a mixture of independent and constrained refinement
*S* = 1.10	*w* = 1/[σ^2^(*F*_o_^2^) + (0.0518*P*)^2^ + 5.0006*P*] where *P* = (*F*_o_^2^ + 2*F*_c_^2^)/3
3702 reflections	(Δ/σ)_max_ = 0.001
180 parameters	Δρ_max_ = 0.45 e Å^−^^3^
0 restraints	Δρ_min_ = −0.30 e Å^−^^3^

### Special details

**Table:**

Experimental. The crystal was placed in the cold stream of an Oxford Cryosystems Cobra open-flow nitrogen cryostat (Cosier & Glazer, 1986) operating at 100.0 (1) K.
Geometry. All e.s.d.'s (except the e.s.d. in the dihedral angle between two l.s. planes) are estimated using the full covariance matrix. The cell e.s.d.'s are taken into account individually in the estimation of e.s.d.'s in distances, angles and torsion angles; correlations between e.s.d.'s in cell parameters are only used when they are defined by crystal symmetry. An approximate (isotropic) treatment of cell e.s.d.'s is used for estimating e.s.d.'s involving l.s. planes.
Refinement. Refinement of *F*^2^ against ALL reflections. The weighted *R*-factor *wR* and goodness of fit *S* are based on *F*^2^, conventional *R*-factors *R* are based on *F*, with *F* set to zero for negative *F*^2^. The threshold expression of *F*^2^ > σ(*F*^2^) is used only for calculating *R*-factors(gt) *etc*. and is not relevant to the choice of reflections for refinement. *R*-factors based on *F*^2^ are statistically about twice as large as those based on *F*, and *R*- factors based on ALL data will be even larger.

### Fractional atomic coordinates and isotropic or equivalent isotropic displacement parameters (Å^2^)

**Table:**

	*x*	*y*	*z*	*U*_iso_*/*U*_eq_	
N1	0.86599 (8)	0.70451 (8)	0.73009 (5)	0.0194 (2)	
N2	0.83258 (9)	0.72622 (8)	0.83600 (6)	0.0248 (3)	
C1	0.88264 (9)	0.65309 (9)	0.67916 (7)	0.0225 (3)	
H1A	0.8954	0.6788	0.6392	0.027*	
C2	0.88132 (10)	0.56542 (9)	0.68453 (7)	0.0235 (3)	
H2A	0.8935	0.5299	0.6488	0.028*	
C3	0.86144 (9)	0.52768 (8)	0.74423 (6)	0.0196 (3)	
C4	0.84480 (9)	0.58047 (8)	0.79506 (6)	0.0196 (3)	
H4A	0.8312	0.5558	0.8352	0.024*	
C5	0.84777 (9)	0.67176 (9)	0.78816 (6)	0.0186 (3)	
C6	0.86067 (10)	0.43092 (9)	0.75102 (7)	0.0247 (3)	
H6A	0.8378	0.4153	0.7929	0.037*	
H6B	0.9198	0.4086	0.7467	0.037*	
H6C	0.8239	0.4056	0.7179	0.037*	
O1	0.53366 (7)	0.62983 (8)	0.91513 (5)	0.0265 (2)	
O2	0.87586 (6)	0.63112 (7)	1.05329 (5)	0.0220 (2)	
O3	0.85543 (7)	0.62684 (8)	0.94864 (5)	0.0278 (3)	
C7	0.67779 (8)	0.63135 (8)	0.95960 (6)	0.0174 (3)	
H7A	0.7030	0.6265	0.9186	0.021*	
C8	0.58817 (9)	0.63434 (9)	0.96604 (6)	0.0190 (3)	
C9	0.55143 (9)	0.64153 (10)	1.02625 (7)	0.0236 (3)	
H9A	0.4903	0.6436	1.0308	0.028*	
C10	0.60424 (9)	0.64573 (10)	1.07952 (6)	0.0234 (3)	
H10A	0.5789	0.6507	1.1205	0.028*	
C11	0.69395 (9)	0.64269 (9)	1.07361 (6)	0.0201 (3)	
H11A	0.7297	0.6455	1.1103	0.024*	
C12	0.73072 (8)	0.63549 (8)	1.01339 (6)	0.0163 (2)	
C13	0.82751 (8)	0.63125 (9)	1.00454 (6)	0.0182 (3)	
H1O1	0.5651 (14)	0.6250 (13)	0.8797 (10)	0.044 (6)*	
H1N1	0.8684 (13)	0.7633 (14)	0.7223 (10)	0.043 (6)*	
H1N2	0.8428 (13)	0.7814 (13)	0.8286 (9)	0.036 (5)*	
H2N2	0.8309 (12)	0.7032 (13)	0.8744 (9)	0.035 (5)*	

### Atomic displacement parameters (Å^2^)

**Table:**

	*U*^11^	*U*^22^	*U*^33^	*U*^12^	*U*^13^	*U*^23^
N1	0.0252 (6)	0.0171 (5)	0.0158 (6)	0.0003 (4)	0.0020 (4)	0.0011 (4)
N2	0.0410 (7)	0.0191 (6)	0.0144 (6)	−0.0007 (5)	0.0048 (5)	0.0002 (5)
C1	0.0297 (7)	0.0241 (7)	0.0137 (6)	0.0018 (5)	0.0038 (5)	0.0017 (5)
C2	0.0317 (7)	0.0227 (7)	0.0159 (7)	0.0032 (5)	0.0036 (5)	−0.0013 (5)
C3	0.0214 (6)	0.0179 (6)	0.0194 (7)	0.0010 (5)	−0.0018 (5)	0.0004 (5)
C4	0.0242 (6)	0.0192 (6)	0.0155 (6)	−0.0011 (5)	0.0007 (5)	0.0021 (5)
C5	0.0201 (6)	0.0204 (6)	0.0154 (6)	−0.0001 (5)	0.0009 (5)	0.0012 (5)
C6	0.0358 (8)	0.0167 (6)	0.0217 (7)	0.0006 (5)	−0.0009 (6)	0.0003 (5)
O1	0.0195 (5)	0.0455 (7)	0.0145 (5)	0.0015 (4)	−0.0043 (4)	−0.0043 (4)
O2	0.0178 (4)	0.0327 (5)	0.0154 (5)	0.0010 (4)	−0.0015 (4)	−0.0004 (4)
O3	0.0201 (5)	0.0490 (7)	0.0143 (5)	0.0031 (4)	0.0022 (4)	0.0033 (4)
C7	0.0194 (6)	0.0199 (6)	0.0129 (6)	0.0005 (5)	0.0007 (5)	−0.0008 (5)
C8	0.0197 (6)	0.0226 (6)	0.0147 (6)	0.0004 (5)	−0.0028 (5)	−0.0024 (5)
C9	0.0164 (6)	0.0354 (8)	0.0189 (7)	0.0000 (5)	0.0004 (5)	−0.0041 (6)
C10	0.0205 (6)	0.0371 (8)	0.0127 (6)	0.0008 (5)	0.0025 (5)	−0.0033 (5)
C11	0.0189 (6)	0.0288 (7)	0.0128 (6)	−0.0004 (5)	−0.0018 (5)	−0.0014 (5)
C12	0.0168 (6)	0.0176 (6)	0.0146 (6)	0.0005 (4)	−0.0001 (4)	0.0010 (4)
C13	0.0168 (6)	0.0220 (6)	0.0157 (6)	0.0007 (5)	0.0007 (5)	0.0015 (5)

### Geometric parameters (Å, º)

**Table:**

N1—C5	1.3521 (17)	C6—H6C	0.9800
N1—C1	1.3582 (18)	O1—C8	1.3641 (16)
N1—H1N1	0.92 (2)	O1—H1O1	0.89 (2)
N2—C5	1.3326 (18)	O2—C13	1.2686 (16)
N2—H1N2	0.88 (2)	O3—C13	1.2546 (16)
N2—H2N2	0.88 (2)	C7—C8	1.3914 (18)
C1—C2	1.359 (2)	C7—C12	1.3975 (18)
C1—H1A	0.9500	C7—H7A	0.9500
C2—C3	1.4184 (19)	C8—C9	1.3924 (19)
C2—H2A	0.9500	C9—C10	1.3876 (19)
C3—C4	1.3691 (19)	C9—H9A	0.9500
C3—C6	1.5012 (19)	C10—C11	1.3918 (19)
C4—C5	1.4180 (18)	C10—H10A	0.9500
C4—H4A	0.9500	C11—C12	1.3929 (18)
C6—H6A	0.9800	C11—H11A	0.9500
C6—H6B	0.9800	C12—C13	1.5078 (18)
			
C5—N1—C1	122.25 (12)	H6A—C6—H6C	109.5
C5—N1—H1N1	122.5 (13)	H6B—C6—H6C	109.5
C1—N1—H1N1	115.3 (14)	C8—O1—H1O1	108.9 (14)
C5—N2—H1N2	116.4 (13)	C8—C7—C12	120.09 (12)
C5—N2—H2N2	116.2 (13)	C8—C7—H7A	120.0
H1N2—N2—H2N2	123.9 (18)	C12—C7—H7A	120.0
N1—C1—C2	120.95 (13)	O1—C8—C7	122.39 (12)
N1—C1—H1A	119.5	O1—C8—C9	117.83 (12)
C2—C1—H1A	119.5	C7—C8—C9	119.77 (12)
C1—C2—C3	119.10 (13)	C10—C9—C8	119.93 (13)
C1—C2—H2A	120.5	C10—C9—H9A	120.0
C3—C2—H2A	120.5	C8—C9—H9A	120.0
C4—C3—C2	119.20 (12)	C9—C10—C11	120.76 (13)
C4—C3—C6	121.12 (12)	C9—C10—H10A	119.6
C2—C3—C6	119.67 (12)	C11—C10—H10A	119.6
C3—C4—C5	120.39 (12)	C10—C11—C12	119.32 (12)
C3—C4—H4A	119.8	C10—C11—H11A	120.3
C5—C4—H4A	119.8	C12—C11—H11A	120.3
N2—C5—N1	118.89 (12)	C11—C12—C7	120.12 (12)
N2—C5—C4	122.99 (12)	C11—C12—C13	121.33 (12)
N1—C5—C4	118.11 (12)	C7—C12—C13	118.55 (12)
C3—C6—H6A	109.5	O3—C13—O2	123.75 (12)
C3—C6—H6B	109.5	O3—C13—C12	117.31 (12)
H6A—C6—H6B	109.5	O2—C13—C12	118.93 (12)
C3—C6—H6C	109.5		
			
C5—N1—C1—C2	0.2 (2)	O1—C8—C9—C10	179.56 (13)
N1—C1—C2—C3	0.6 (2)	C7—C8—C9—C10	0.0 (2)
C1—C2—C3—C4	−0.5 (2)	C8—C9—C10—C11	−0.1 (2)
C1—C2—C3—C6	−179.36 (14)	C9—C10—C11—C12	0.1 (2)
C2—C3—C4—C5	−0.3 (2)	C10—C11—C12—C7	0.0 (2)
C6—C3—C4—C5	178.53 (13)	C10—C11—C12—C13	−179.53 (13)
C1—N1—C5—N2	179.63 (13)	C8—C7—C12—C11	0.0 (2)
C1—N1—C5—C4	−1.0 (2)	C8—C7—C12—C13	179.47 (12)
C3—C4—C5—N2	−179.66 (14)	C11—C12—C13—O3	−178.37 (13)
C3—C4—C5—N1	1.0 (2)	C7—C12—C13—O3	2.12 (19)
C12—C7—C8—O1	−179.46 (12)	C11—C12—C13—O2	2.58 (19)
C12—C7—C8—C9	0.0 (2)	C7—C12—C13—O2	−176.92 (12)

### Hydrogen-bond geometry (Å, º)

**Table:**

*D*—H···*A*	*D*—H	H···*A*	*D*···*A*	*D*—H···*A*
O1—H1*O*1···O2^i^	0.89 (2)	1.81 (2)	2.6970 (15)	172 (2)
N1—H1*N*1···O2^ii^	0.92 (2)	1.81 (2)	2.7221 (15)	172 (2)
N2—H1*N*2···O3^ii^	0.88 (2)	1.91 (2)	2.7852 (16)	170.6 (19)
N2—H2*N*2···O3	0.883 (19)	1.994 (19)	2.8454 (17)	161.7 (18)

Symmetry codes: (i) −*y*+5/4, *x*−1/4, *z*−1/4; (ii) *y*+1/4, −*x*+7/4, −*z*+7/4.

## References

[bb1] Allen, F. H., Kennard, O., Watson, D. G., Brammer, L., Orpen, A. G. & Taylor, R. (1987). *J. Chem. Soc. Perkin Trans. 2*, pp. S1–19.

[bb2] Bernstein, J., Davis, R. E., Shimoni, L. & Chang, N.-L. (1995). *Angew. Chem. Int. Ed. Engl.* **34**, 1555–1573.

[bb3] Bruker (2009). *SADABS*, *APEX2* and *SAINT* Bruker AXS Inc., Madison, Wisconsin, USA.

[bb4] Cosier, J. & Glazer, A. M. (1986). *J. Appl. Cryst.* **19**, 105–107.

[bb5] Goswami, S. & Ghosh, K. (1997). *Tetrahedron Lett.* **38**, 4503–4506.

[bb6] Goswami, S., Mahapatra, A. K., Nigam, G. D., Chinnakali, K. & Fun, H.-K. (1998). *Acta Cryst.* C**54**, 1301–1302.

[bb7] Kvick, Å. & Noordik, J. (1977). *Acta Cryst.* B**33**, 2862–2866.

[bb8] Lehn, J. M. (1992). *J. Coord. Chem.* **27**, 3–6.

[bb9] Sheldrick, G. M. (2008). *Acta Cryst.* A**64**, 112–122. 10.1107/S0108767307043930 18156677

[bb10] Spek, A. L. (2009). *Acta Cryst.* D**65**, 148–155. 10.1107/S090744490804362X PMC2631630 19171970

